# Predicting Irida-Silicene: A Novel 2D Silicon Allotrope

**DOI:** 10.1021/acsomega.4c08395

**Published:** 2024-12-10

**Authors:** Djardiel da S. Gomes, Luiz A. Ribeiro, Marcelo L. Pereira

**Affiliations:** † Faculty UnB Planaltina, Materials Science Postgraduate Program, University of Brasília, Brasília, Federal District 73345-010, Brazil; ‡ Institute of Physics, University of Brasília, Brasília, Federal District 04455, Brazil; § Computational Materials Laboratory, University of Brasília, Brasília, Federal District 70910-900, Brazil; ∥ Department of Electrical Engineering, College of Technology, University of Brasília, Brasília, Federal District 70910-900, Brazil

## Abstract

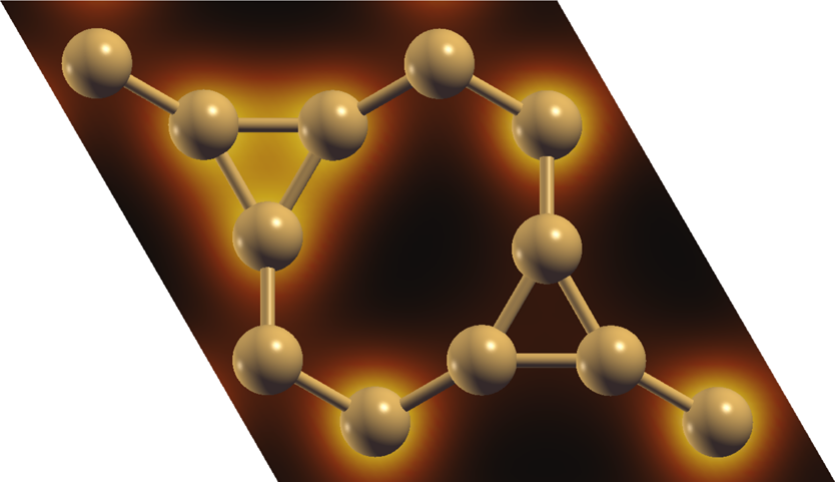

Two-dimensional (2D) silicon-based materials have garnered significant attention for their promising properties, making them suitable for various advanced technological applications. Here, we present Irida-Silicene (ISi), a novel 2D silicon allotrope inspired by Irida-Graphene (IG), which was recently proposed and is entirely composed of carbon atoms. ISi exhibits a buckled structure composed of 3–6–8 membered rings, unlike its planar carbon counterpart. Using density functional theory (DFT) calculations, we discuss its stability, structural, electronic, optical, and mechanical properties. Our results indicate that ISi exhibits bond lengths ranging from 2.27 to 2.32 Å, with buckling of 0.78 Å, the latter significantly larger than that reported for silicene. The nanomaterial demonstrates good dynamical and thermal stability at room temperature, without phonon dispersion with imaginary frequencies, and a cohesive energy of −4.98 eV/atom. ISi is a metallic monolayer with a Dirac cone above the Fermi level in the center of the band, and it is also a nonmagnetic material. Furthermore, the system displays anisotropic electronic properties, showing semiconducting behavior depending on the direction, with a region devoid of electronic states between −0.2 and −0.8 eV. The optical activity of ISi is primarily observed in the infrared and ultraviolet regions, with a peak for photons with an energy of 5.5 eV in the latter case. Finally, regarding mechanical properties, we report estimated elastic and bulk moduli of approximately 34 and 41 N/m, respectively. The system can withstand up to 15% of strain, depending on the direction and type of deformation. These findings suggest that ISi holds potential for various technological applications, expanding the potential uses of 2D silicon-based materials beyond silicene.

## Introduction

Silicon-based technologies form the backbone of modern electronics, enabling a diverse array of devices, including microprocessors^1,2^ and solar cells.^3,4^ Advancements in these technologies depend on discovering and developing novel materials capable of improving the performance and functionality of electronic and optoelectronic systems.^5,6^ Among these, two-dimensional (2D) silicon-based materials have become a prominent area of research since the breakthrough in silicene synthesis,^7−10^ offering unique characteristics absent in their bulk forms.

Silicene, a monolayer of silicon atoms organized in a hexagonal lattice reminiscent of graphene, has garnered significant attention within the scientific community.^11−15^ With properties such as a tunable bandgap and remarkable carrier mobility,^16^ silicene is regarded as a promising material for nanoscale applications, including sensors,^17^ transistors,^18^ and other miniaturized devices.^11^ The theoretical predictions and experimental achievements surrounding silicene have paved the way for exploring silicon-based 2D materials in cutting-edge technologies.^11^

Recent advancements in material synthesis techniques have enabled the development of silicene-based materials, broadening their applicability across diverse technological domains.^19^ Density Functional Theory (DFT) has played a crucial role in unveiling various silicene-based structures’ existence, stability, and properties. This computational approach has provided valuable insights into their electronic, mechanical, and optical behaviors.^20,21^ These theoretical findings have, in turn, driven experimental research to leverage silicene’s unique potential and its derivatives.^22^

A novel 2D carbon allotrope known as Irida-Graphene (IG)^23^ was recently introduced in the literature. This planar 2D nanomaterial consists of carbon atoms arranged in a unique pattern of 3–6–8 membered rings. IG has garnered considerable attention within the scientific community, prompting extensive research to characterize its properties and assess its potential applications.^24−30^ Despite the growing interest, most investigations have concentrated on exploring new carbon-based nanostructures. In this study, we present Irida-Silicene (ISi), a silicon-based analog of IG. While IG is recognized for its promising attributes across diverse applications, our research focuses on the silicon counterpart, aiming to leverage silicon’s advantageous properties while uncovering novel characteristics arising from its distinctive structural configuration.

The synthesis of 2D materials has been a central research focus in materials science for several decades,^31^ gaining renewed momentum following the exfoliation of graphene nearly 20 years ago.^32^ Recent years have witnessed significant progress, particularly in synthesizing carbon-based 2D materials.^33−36^ For Irida-Si, chemical vapor deposition (CVD), mechanical exfoliation, and liquid-phase exfoliation offer promising pathways for successful fabrication.^31^ Developing small silicon-based clusters^37^ presents another intriguing avenue. Among the most promising approaches, the progress in synthesizing silicon-based biphenylene networks^22^ could serve as a critical stepping stone toward realizing Irida-Si.

In this work, we employ DFT calculations to predict and analyze the properties of ISi. We investigate its stability and structural, electronic, optical, and mechanical properties to understand its potential for practical applications. Our results reveal that ISi demonstrates good dynamical and thermal stability, a significant elastic modulus, and electronic and optical properties of interest for various new advanced technologies.

## Methodology

The geometry optimizations, structural stability, and the optical and electronic properties of ISi were analyzed using first-principles calculations implemented in the SIESTA code,^38,39^ within the DFT framework.^40,41^ The generalized gradient approximation (GGA) was applied with the Perdew–Burke–Ernzerhof (PBE) parametrization.^42^ Recognizing the tendency of GGA/PBE to underestimate band gaps, additional electronic structure calculations were performed using the hybrid Heyd-Scuseria–Ernzerhof functional (HSE06),^43^ implemented via the HONPAS package.^44,45^

The electron–ion interactions were modeled using Troullier–Martins norm-conserving pseudopotentials, considering a 3s^2^3p^2^ valence electron configuration for Si atoms and employing the Kleinman–Bylander separable form.^46,47^ All calculations utilized an energy cutoff of 800 Ry and a double-ζ (DZP) basis set constructed from numerical atomic orbitals with a finite range. For Brillouin zone integration, we employed the Monkhorst–Pack grid with dimensions 50 × 50 × 1.^48^

It is important to note that ultrasoft pseudopotentials (USPPs) are generally more precise for electronic property calculations than norm-conserving pseudopotentials (NCPs), particularly in systems where accurately describing wave functions near the core is critical. However, in this study, NCPs were adequate to capture the primary trends and phenomena of interest without imposing significant additional computational demands. Furthermore, we validated the reliability of the chosen pseudopotentials by comparing benchmarked results from the literature, including experimental data and theoretical studies, confirming their suitability for silicon-based systems. While USPPs could provide finer accuracy, using NCPs represents an optimal compromise between computational efficiency and the level of accuracy required for our research objectives.

During optimization, lattice vectors and atomic positions were fully relaxed until the maximum force acting on each atom was below 0.001 eV/Å, and the total energy change was less than 10^–5^ eV. A vacuum thickness of 30 Å was introduced between monolayers to eliminate distortions arising from interlayer interactions.

Phonon calculations were carried out to assess the mechanical stability of ISi structures. These calculations allow for identifying vibrational modes with imaginary (negative) frequencies, which signal dynamical instability in the monolayer. A 3 × 3 × 1 supercell was employed, with phonon dispersion interpolated across the Brillouin zone using a mesh cutoff of 800 Ry. Convergence criteria were set to 10^–5^ for energy and 0.001 eV/Å for force, and the acoustic sum rule was enforced at the Γ point.

Ab Initio Molecular Dynamics (AIMD) simulations were conducted to evaluate thermal stability using a 2 × 2 × 1 supercell containing 48 atoms at 300 K. These simulations used a time step of 0.5 fs within the *NPT* ensemble. Pressure components in the plane were controlled by a Parrinello–Rahman barostat, while a Nosé–Hoover thermostat maintained the system temperature. This approach, frequently employed for studying material stability,^49−51^ involved evolving the system for 10 ps following heating to the target temperature.

We applied a standard external electric field of 1.0 V/Å along the *x*-, *y*-, and *z*-directions individually for optical calculations. The real (ε_1_) and imaginary (ε_2_) parts of the dielectric constant were derived using the Kramers–Kronig relation^52,53^ and Fermi’s golden rule.^54^ The real part of the dielectric constant is given by
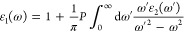
1where *P* denotes the principal value of the integral over ω′. The imaginary part, which accounts for interband optical transitions between the valence band (VB) and the conduction band (CB), is expressed as
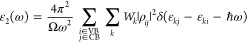
2where ω is the photon frequency, |ρ_*ij*_| is the dipole transition matrix element, and *W*_*k*_ is the weight of the respective *k*-point in the reciprocal space. Additionally, Ω represents the system volume. From ε_1_ and ε_2_, other properties such as the absorption coefficient (α), reflectance (*R*), and refractive index (η) can be determined as follows:
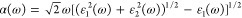
3
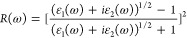
4
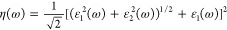
5

After determining the stable ISi structure, we calculated its mechanical strengths. To obtain the mechanical properties of two-dimensional materials, we typically calculate the in-plane stiffness, Poisson’s ratio, and bulk modulus values. For ISi, we used a supercell (2 × 2 × 1) in the *xy* plane and applied tension along the *x*, *y*, and *xy* directions separately. For uniaxial strain, the lattice constants, *a* and *b*, were varied in increments of 0.5% until the end of the system’s plastic region, starting from their optimized values. The energy for each strain step was calculated. Based on these calculations, the in-plane stiffness is expressed as^55,56^
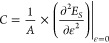
6where *A* represents the area of the optimized supercell, ε is the strain due to uniaxial deformation, defined as ε = Δ*l*/*l*_0_, with *l*_0_ being the supercell length in the equilibrium position, and Δ*l* = *l* – *l*_0_ representing the change in length after applying the strain. Finally, *E*_S_ corresponds to the strain energy, calculated at each point by subtracting the total energy at that point from the total equilibrium energy, i.e., the energy without any applied deformation.

On the other hand, the in-plane bulk modulus is calculated as the second derivative of the total strain energy concerning the area of the two-dimensional nanostructures:
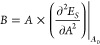
7where *A*_0_ and *A* represent the unit cell area in its equilibrium position (without any strain) and after applying strain, respectively.^57,58^

Finally, Poisson’s ratio was also calculated for IG, obtained from the ratio of transverse strain (ε_trans_) to axial strain (ε_axial_) for small strain values, as shown below:^59^
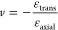
8

## Results and Discuss

**Figure 1:**
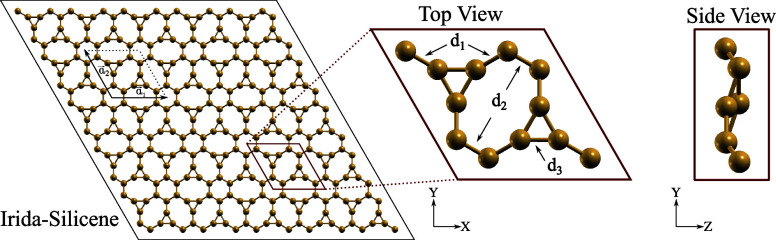
Schematic representation of the ISi structure is shown in three panels. The leftmost panel provides a top view of the material, illustrating its lattice vectors *a*_1_ = *a*_2_ = 9.81 Å. The middle panel zooms in on the unit cell, and the rightmost panel presents a lateral view, highlighting the material’s buckling.

We begin by analyzing the optimized lattice structure of ISi, as shown in Figure 1. The bond lengths in the nanomaterial are categorized into three distinct values in ISi, denoted as d_1_, corresponding to bonds exclusive to the eight-membered rings, d_2_, representing bonds that connect octagonal rings with hexagons, and d_3_, denoting bonds within the silicon triangles in the system, and are found to be 2.29, 2.32, and 2.27 Å, respectively. These measurements are consistent with the bond lengths observed in silicene, which typically range almost 2.3 Å.^60^ Finally, the density of ISi is 6.7 × 10^–4^ g/m^2^, while the density of silicene is approximately 7.2 × 10^–4^ g/m^2^, resulting in a ratio of approximately 0.93. The buckled nature of ISi is evident, with a vertical displacement of approximately 0.78 Å, which is greater than the approximately 0.44 Å observed in silicene.^61^ This buckling arises from the sp^3^ hybridization of silicon atoms, distinguishing ISi from planar sp^2^ hybridized materials such as graphene.^62^ The pronounced buckling in ISi confirms its adherence to the known structural characteristics of silicene while incorporating distinct features from its IG counterpart.^23^

The lattice parameters of ISi are *a*_1_ = *a*_2_ = 9.81 Å, resulting in a trigonal crystal structure with the *P*-3*M*1 space group. Although ISi deviates from the hexagonal symmetry of silicene, it maintains comparable bond lengths, demonstrating the adaptability of silicon–silicon bonds in various structural arrangements. The cohesive energy (*E*_coh_) of ISi is determined by the equation *E*_coh_ = (*E*_ISi_ – *N*·*E*_Si_)/*N*, where *E*_ISi_ represents the total energy of the ISi structure, *N* is the number of silicon atoms in unit cell, and *E*_Si_ is the energy of a single silicon atom. Here, the energy of a single isolated silicon atom is calculated as *E*_Si_ = −122.88 eV. The formation energy of ISi is estimated to be *E*_coh_ = −4.98 eV/atom, indicating that the material is energetically favorable and potentially synthesizable under suitable conditions. The same protocol was applied to silicene for comparison, yielding *E*_coh_ = −5.25 eV/atom. As expected, the system with a hexagonal arrangement is more stable than ISi. The formation energies obtained also agree with those found for other two-dimensional silicon allotropes reported in the literature.^63−65^ The favorable formation energy emphasizes the potential stability of ISi.

**Figure 2:**
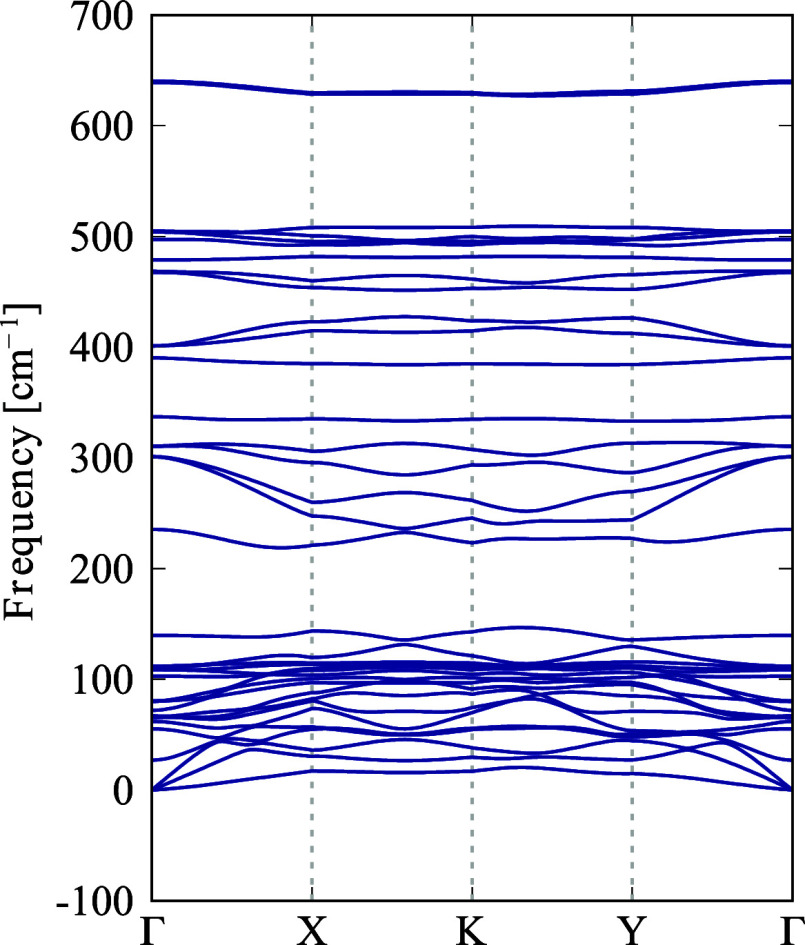
Phonon band structure of ISi with a 3 × 3 × 1 supercell.

As previously discussed, the thermal and dynamical stability of ISi was evaluated using AIMD simulations and phonon dispersion analysis. The phonon dispersion curves for ISi, shown in Figure 2, reveal no imaginary frequencies across the Brillouin zone. The lack of imaginary frequencies confirms that ISi is dynamically stable and resistant to minor perturbations.

The phonon dispersion curves of ISi reveal no band gap between the acoustic and optical modes, with the highest phonon frequency reaching approximately 640 cm^–1^. This value is slightly higher than the 562 cm^–1^ observed for silicene,^66^ but significantly lower than the 1600 cm^–1^ reported for IG.^23^ The phonon frequencies of ISi and silicene are comparable, suggesting similar vibrational characteristics despite their structural differences. The overlap between the acoustic and optical phonon bands in ISi indicates strong intermode coupling, a typical feature of silicon-based 2D materials.^64,67^ This coupling is advantageous for applications related to thermal conductivity, implying that ISi may have efficient heat dissipation properties. These results emphasize the stability of ISi and its potential for applications where effective thermal management is essential. The observed phonon behavior aligns with the expected properties of silicon-based 2D materials,^64,68^ reinforcing ISi’s suitability for further experimental investigation and practical applications.

**Figure 3:**
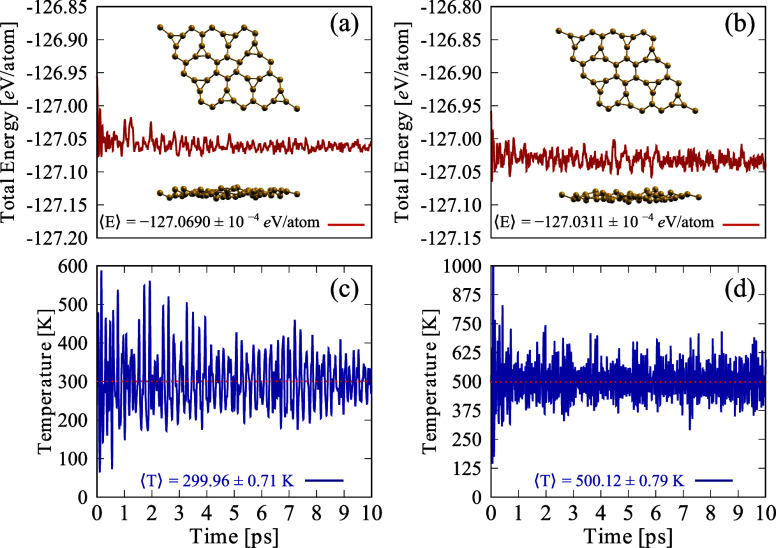
Temporal evolution of (a, b) the total energy and (c, d) system temperature over the simulation period for the external temperature of 300 K (a, c) and 500 K (b, d). The insets in panel (a) show the top and side views of the final AIMD snapshot at 10 ps.

To further evaluate the thermal stability of ISi, AIMD simulations were performed at room temperature. Figure 3a shows the evolution of the total energy over the 10 ps simulation period, which remains nearly constant with only slight fluctuations. This behavior indicates that ISi demonstrates significant thermal stability. The inset images in Figure 3a show both top and side views of the final AIMD configuration. Although some deformation is observed in the ISi structure at this temperature, no bond rupture occurs, and the overall structure remains stable. The deformations are mainly attributed to slight changes in planarity and minor variations in bond lengths between the lattice atoms, which are typical under thermal stress. Figure 3c illustrates the time evolution of the system temperature. One can note that the average temperature is 300 K. Figure 3b,d presents the same analysis conducted at room temperature, now at 500 K. It is important to note that the thermal variation of the system is more significant, with ISi being subjected to temperatures close to 800 K at various instances, demonstrating that ISi is stable at temperatures commonly used for the synthesis of these two-dimensional materials. Although synthesis processes may occur above this temperature range, we aim to present that the topology of ISi can withstand temperature increases consistent with applications in electronics and other technologies. In all cases, the error presented in Figure 3 is obtained from the uncertainty of the average value, given by , where σ is the standard deviation and *N* is the sample size, respectively.

**Figure 4:**
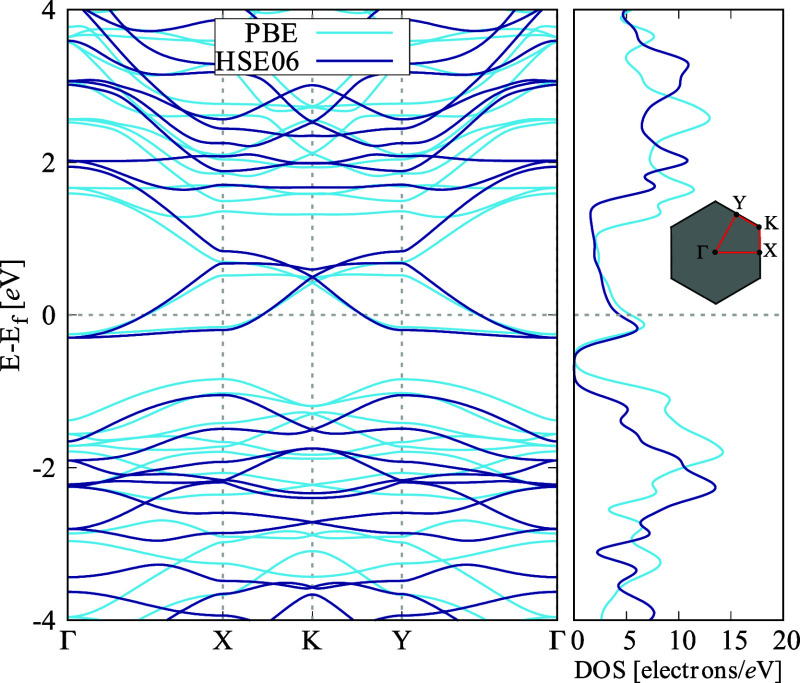
Electronic band structure and related density of states (DOS) of ISi. We considered the following path along the high symmetry directions: Γ = (0, 0) to *K* = (0, 1/2) to *Y* = (1/3, 1/3) to *Z* = (1/2, 0) to Γ = (0, 0).

The electronic characteristics of ISi were thoroughly investigated to gain a comprehensive understanding of its conductivity, electronic structure, and electron behavior. The electronic band structure and corresponding density of states (DOS) are depicted in Figure 4. The analysis shows that ISi does not exhibit a band gap between the valence and conduction bands, classifying it as a metallic material, similar to IG.^23^ This metallic nature is particularly apparent along the *X*–*Y* direction in the Brillouin zone. However, an interesting feature of ISi is its anisotropic electronic behavior. Along the *Y*–Γ path, the material demonstrates semiconducting properties. The DOS further supports this observation, as there is a noticeable absence of electronic states in the energy range between −0.2 and −0.8 eV. This anisotropy in electronic conduction is likely due to the unique structural elements of ISi, especially its buckled geometry and the distinctive ring-like arrangement within the lattice. These specific ring motifs create favored routes for electron movement, resulting in directionally dependent electrical conductivity.

The band structure also reveals a linear energy dispersion near the Fermi level, particularly near the *X*–*K* and *K*–*Y* points in the reciprocal space. This linear dispersion suggests that charge carriers in ISi behave similarly to massless Dirac Fermions, which indicates high electronic mobility—a crucial property for applications in fast electronics.

**Figure 5:**
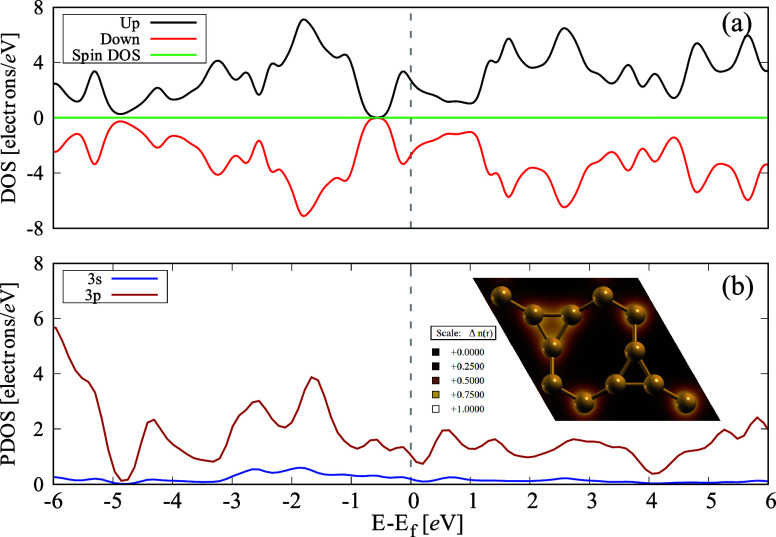
(a) ISi DOS for electrons with spin up (black) and spin down (red), along with the spin DOS (green) and related (b) partial density of states (PDOS) and ELF (inset panel).

Figure 5a displays the DOS for spin-up (black) and spin-down (red) electrons, along with the total spin DOS (green). The symmetry of the spin-up and spin-down DOS suggests that the ISi structure does not exhibit magnetization, with the total spin DOS effectively zero. Additionally, Figure 5b shows the partial density of states (PDOS) and the electron localization function (ELF, inset). The states at the Fermi level are primarily formed from Si-3p orbitals, with no significant contribution from Si-3s orbitals. This behavior implies that the 3p orbitals are the critical contributors to the electronic behavior and interactions in ISi, reinforcing its classification as a metallic material.

Similarities and distinct differences become evident when comparing ISi to silicene and other 2D silicon allotropes. Like silicene, ISi exhibits metallic behavior, with the electronic structure near the Fermi level primarily influenced by Si-3p orbitals.^69,70^ However, a notable distinction lies in the anisotropic conductance of ISi, which is more pronounced than silicene, where the electronic properties tend to be more isotropic. Despite this, the linear energy dispersion observed near the Fermi level in both materials suggests they exhibit high carrier mobility, which is essential for potential applications in high-speed electronics.

To gain deeper insights into the bonding interactions within ISi, we analyzed the ELF. Figure 5b shows the ELF, providing a topological view of electron distribution in the material. The ELF values span from 0.0 to 1.0, where values closer to 1.0 indicate strong covalent bonds or lone pair electrons. In contrast, values around 0.75 suggest electron delocalization, typical of metallic bonding or weak van der Waals forces. The ELF analysis reveals regions of substantial electron localization, signifying covalent solid interactions. Notably, the areas with ELF values near 0.75 highlight the robust covalent bonding between silicon atoms forming the three-atom rings. Additionally, intense yellow regions are visible in the octagonal ring atoms, where strong bonding occurs with atoms in the three-atom rings. These localized electron interactions are pivotal to the structural stability and rigidity of ISi, significantly contributing to the material’s mechanical properties.

In the bond network of ISi, the silicon atoms in the hexagonal and octagonal rings exhibit lighter yellow regions with ELF values below 0.5, indicative of more delocalized electron density. This delocalization points to metallic-like behavior in these areas, which is consistent with the overall metallic nature of ISi. The simultaneous presence of localized and delocalized electrons in ISi’s bonding structure contributes to its anisotropic conductance, as reflected in its electronic band structure. Materials featuring delocalized valence electrons typically show metallic conductivity, whereas those with robust covalent bonding often behave as semiconductors. ISi’s hybrid bonding nature thus imparts a blend of metallic and semiconductor-like electronic properties, enabling high electrical conductivity and directional electronic behavior. This dual nature of bonding is crucial for the potential use of ISi in a wide range of electronic and optoelectronic applications, combining the strengths of both types of materials.

Its optical properties were analyzed to investigate ISi’s potential for optoelectronic applications, providing further insight into its electronic structure. Initially, the real and imaginary parts of the dielectric function of ISi were computed, considering light polarization along the *x*, *y* (in-plane), and *z* (out-of-plane) directions (see Figure S1). Figure S2 shows that, within the plane, the optical properties of ISi are highly isotropic concerning the *x* (100) and *y* (010) directions. Furthermore, when light is polarized along the *z* (001) direction, all in-plane optical properties maintain a similar shape, although with lower intensity.

**Figure 6:**
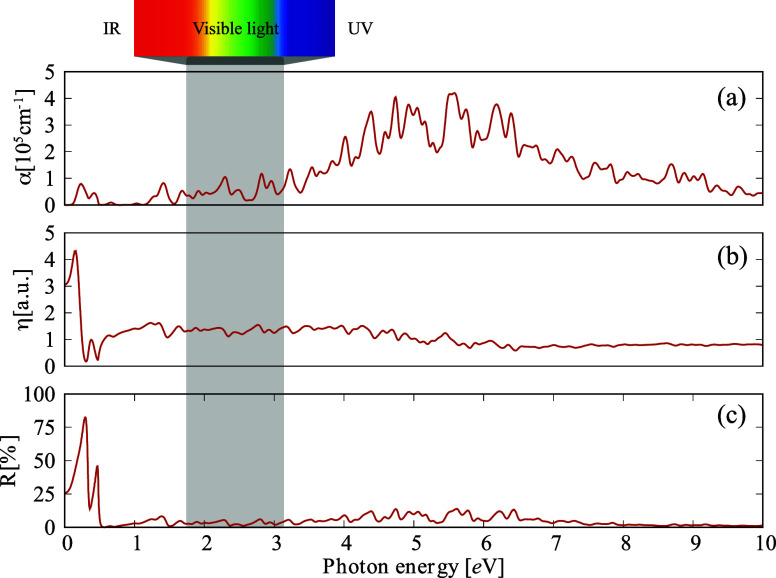
(a) Optical absorption, (b) refractive index, and (c) reflectivity index calculated for a polarized light oriented along the *z*-direction, i.e., perpendicular to the ISi surface.

Figure 6 presents the optical characteristics of ISi for light polarization along the *z* direction, which is of particular interest since the light incidence is perpendicular to the material’s plane. In Figure 6a, the absorption coefficient of ISi is shown to reach values as high as 10^5^ cm^–1^, a consequence of its metallic nature. The most prominent absorption peaks, which occur for light polarized in the *z* direction, are located in the ultraviolet (UV) spectrum, with a peak of around 5.5 eV. This shift to higher energy compared to the absorption peak of silicene (approximately 2.9 eV) suggests that ISi undergoes unique electronic transitions due to its distinct structural and electronic properties. Additionally, the presence of multiple absorption peaks for photon energies above 4.0 eV highlights the potential of ISi for use as a UV detector and absorber, similar to silicene^71^ and other 2D materials composed of various elements.^72,73^

When light is polarized perpendicular to its basal plane, ISi demonstrates refractive activity in the infrared range, as depicted in Figure 6b. This results in ISi being nearly opaque at lower photon energies. Additionally, Figure 6c shows that about 75% of infrared light is reflected, which suggests that ISi could function as an efficient infrared shield, similar to the behavior observed in silicene.^74^ For photon energies exceeding 1.0 eV, the material exhibits a low reflectivity and a refractive index of 1, indicating that incident light in the UV–visible spectrum is transmitted efficiently.

**Figure 7:**
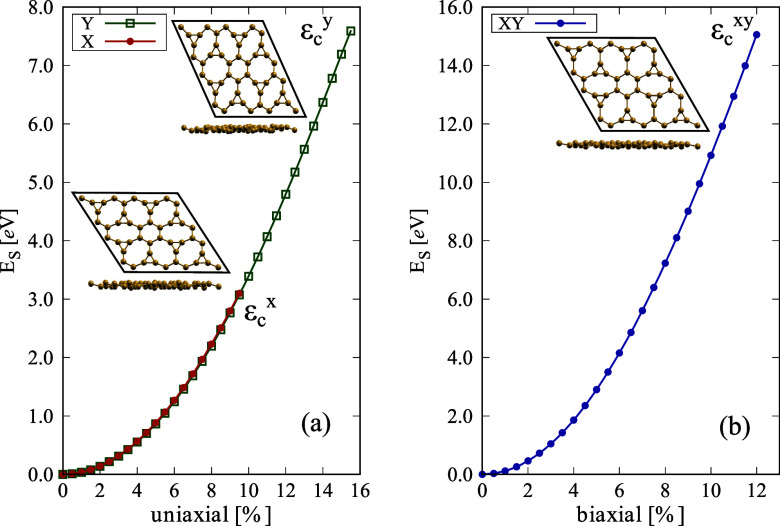
Stress–strain relationships of ISi under (a) uniaxial tensile loading in (orange) the *x*-direction and (green) *y*-direction and (b) biaxial tensile loading.

Finally, the mechanical properties of ISi were examined through uniaxial and biaxial tensile tests, revealing important information about its structural integrity and resilience. Figure 7 presents the energy-strain relationship for tensile loading in the *x* and *y* directions (a) and for biaxial *xy* strain (b). The inset panels illustrate the ISi topology at the critical strain (ε_C_), i.e., the strain value before ISi rupture. The lateral view of the ISi at this point is also presented. The tensile force does not exceed the sp^3^ bond strength, maintaining buckling throughout the deformation process. The energy-strain curves highlight the anisotropic mechanical behavior of ISi. The material demonstrates distinct responses to strain in the *x* and *y* directions, with in-plane stiffness values of *C*_*x*_ = 33.94 and *C*_*y*_ = 33.13 N/m, respectively. Considering the silicene thickness of 4.2 Å and adjusting due to the more significant buckling of ISi, these values correspond to Young’s modulus of *Y*_*x*_ = 74.92 and *Y*_*y*_ = 73.13 GPa in the *x* and *y* directions, respectively. These values are about 55% relative to Silicene, which also exhibits isotropy in the *x* and *y* directions concerning Young’s modulus.^75^ Compared to carbon-based systems, Young’s modulus of IG is around 40% compared to graphene^23^ but with high isotropy, similar to ISi. However, Poisson’s ratio, which measures the material’s tendency to expand perpendicularly to the applied strain, exhibits considerable anisotropy. ISi shows a Poisson’s ratio of 0.06 in the *x* direction and 0.53 in the *y* direction. These values differ significantly, indicating varied deformation mechanisms along different crystallographic directions, further showing that in the *y* direction, ISi is a ductile system (*v* > 0.25).^76^ The Poisson’s ratio calculated here was performed for a strain of 0.5%. The critical strains for ISi are 9.5% in the *x* direction  and 15.5% in the *y* direction , showing considerably higher resilience to strain in the *y* direction, differing from IG, which also showed a high degree of isotropy concerning critical strain.

In the biaxial strain test depicted in Figure of 7b, ISi demonstrates its robustness by maintaining its structural integrity up to a critical strain of 12.0% . The calculated bulk modulus, which measures the material’s resistance to uniform compression in two and three dimensions, is 40.96 N/m. This value indicates that ISi can withstand significant biaxial strain without undergoing rapid failure. The unique ring topology of ISi contributes to its mechanical anisotropy and resilience. The presence of fused rings of three atoms enhances the material’s rigidity. In contrast, the porosity introduced by the eight-atom rings allows for greater flexibility under strain, as pointed by the Posion ratio values. This combination of rigidity and flexibility is reflected in the energy–strain curves, where ISi shows significant elastic deformation before reaching its critical strain thresholds. Compared to silicene, which typically exhibits more isotropic mechanical properties,^75^ ISi’s anisotropic behavior directly results, again, from its unique lattice arrangement.

## Conclusions

In summary, we have thoroughly investigated the properties of Irida-Silicene using DFT calculations and AIMD simulations. Structurally, ISi exhibits bond lengths and formation energy indicative of its potential for experimental synthesis. The stability of ISi was confirmed through AIMD simulations at room temperature, showing no bond breakage, and phonon dispersion analysis, which revealed no imaginary frequencies, thus confirming its dynamic stability.

The electronic properties of ISi demonstrate that it is metallic, with its electronic band structure showing no band gap between the valence and conduction bands. The unique buckled structure and ring topology contribute to its anisotropic conductance. The linear energy dispersions near the Fermi level suggest that charge carriers in ISi can behave like massless Dirac Fermions, highlighting its potential for applications in electronic devices.

Optical property analysis reveals that ISi has a high absorption coefficient in the ultraviolet range, with significant peaks that suggest its use as a UV detector and absorber. The material also displays high reflectivity and refraction within the infrared region, suggesting that it can serve as an effective infrared protector

The mechanical properties of ISi indicate significant anisotropy. In-plane stiffness values of 33.94 N/m in the *x*-direction and 33.13 N/m in the *y*-direction, along with Poisson ratios of 0.06 and 0.53, respectively, demonstrate ISi’s varied deformation responses depending on the direction of the applied strain. The critical strain values of 9.5% in the *x*-direction and 15.5% in the *y*-direction and the bulk modulus of 40.96 N/m under biaxial strain further underscore its mechanical resilience.

The proposition of ISi, characterized as a metallic, nonmagnetic material with an absorption peak in the UV region, along with the aforementioned mechanical properties, opens avenues for various applications, which we encourage to be explored (the CIF file is available in the Supporting Information). Due to its metallic characteristics, it can be considered in terms of charge transport, making it potentially useful as an anode/cathode; thus, studies on charge transport are necessary for a better understanding of the application space of the nanomaterial. Finally, due to its nanoporous nature, featuring eight-atom Si rings, it is suggested that it could be functionalized with metals, as has been done in various studies for its carbon analog,^23^ with a view toward hydrogen storage, lithium-ion batteries, and/or catalysis.

## Supporting Information Available

Crystallographic information file (CIF) for Irida-Silicene and two figures related to the optical properties of ISi; first figure illustrates the real (ε_1_) and imaginary (ε_2_) parts of the dielectric constant as a function of photon energy (Figure S1); and second figure presents the optical properties of ISi when the light is polarized in-plane (Figure S2) (PDF)
