# Supplementary material for: Predicting Irida-Silicene: A Novel 2D Silicon Allotrope
Source: ACS Omega. 2024 Dec 10;9(51):50570–8. doi: 10.1021/acsomega.4c08395 (PMC11683622; doi:10.1021/acsomega.4c08395)
Supplement: Supplementary file 1 — ao4c08395_si_001.pdf [file ao4c08395_si_001.pdf]

Supplementary Information for Predicting Irida-Silicene: A Novel 2D Silicon Allotrope

Djardiel da S. Gomes,[†] Luiz A. Ribeiro Jr,[‡,¶] and Marcelo L. Pereira Jr[*,§,†]

†*University of Brasília, Faculty UnB Planaltina, Materials Science Postgraduate Program, Brasília, Federal District, Brazil.*

‡*University of Brasília, Institute of Physics, Brasília, Federal District, Brazil.*

¶*Computational Materials Laboratory, University of Brasília, Brasília, Federal District, Brazil.*

§*University of Brasília, College of Technology, Department of Electrical Engineering, Brasília, Federal District, Brazil.*

E-mail: marcelo.lopes@unb.br

Contents

1 Crystallographic Information File **S3**

2 Dielectric Function **S5**

3 In-Plane Optical Properties **S6**

1 Crystallographic Information File

ISi.cif

```
data_ISi
_audit_creation_date              2024-08-06
_symmetry_space_group_name_H-M    'P-3M1'
_symmetry_Int_Tables_number       164
_symmetry_cell_setting            trigonal
loop_
_symmetry_equiv_pos_as_xyz
  x,y,z
  -y,x-y,z
  -x+y,-x,z
  y,x,-z
  x-y,-y,-z
  -x,-x+y,-z
  -x,-y,-z
  y,-x+y,-z
  x-y,x,-z
  -y,-x,z
  -x+y,y,z
  x,x-y,z
_cell_length_a                    9.8100
_cell_length_b                    9.8100
_cell_length_c                    30.0000
_cell_angle_alpha                 90.0000
_cell_angle_beta                  90.0000
_cell_angle_gamma                 120.0000
loop_
_atom_site_label
_atom_site_type_symbol
_atom_site_fract_x
```

```
_atom_site_fract_y
_atom_site_fract_z
_atom_site_U_iso_or_equiv
_atom_site_adp_type
_atom_site_occupancy
Si1    Si    0.25615   0.74385   0.48867   0.00000  Uiso   0.00
Si2    Si    0.12843   0.87157   0.51290   0.00000  Uiso   0.00
loop_
_geom_bond_atom_site_label_1
_geom_bond_atom_site_label_2
_geom_bond_distance
_geom_bond_site_symmetry_2
_ccdc_geom_bond_type
Si1    Si2     2.289   .     S
Si1    Si1     2.271   3_565 S
Si1    Si1     2.271   2_665 S
Si2    Si2     2.315   6_556 S
Si2    Si2     2.315   5_676 S
```

2 Dielectric Function

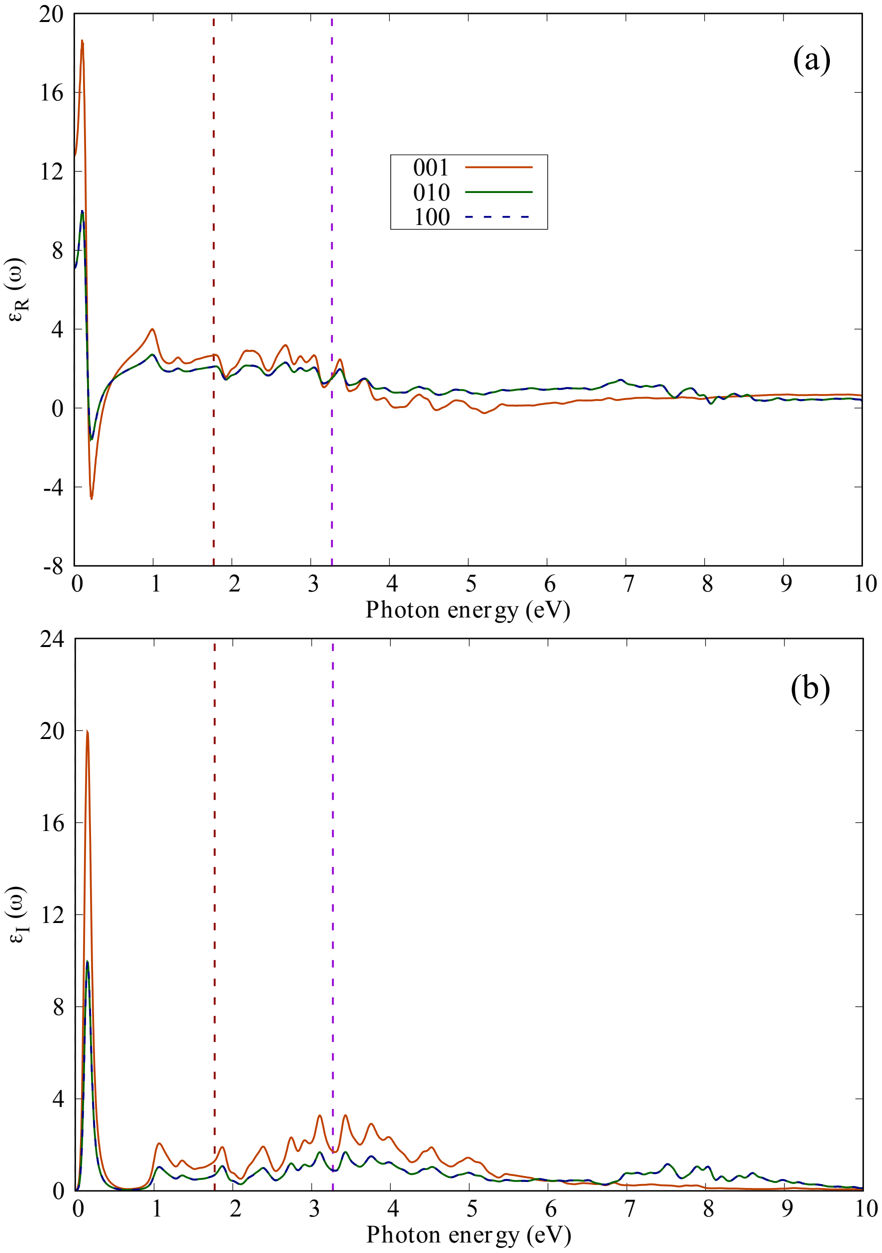

Figure S1: Real (a) and imaginary (b) parts of the dielectric constant for ISi are plotted against photon energy. The real component corresponds to the material's dispersive properties, while the imaginary component relates to its absorptive characteristics. Each peak in the spectrum signifies an optical activity resulting from permissible electronic state transitions.

3 In-Plane Optical Properties

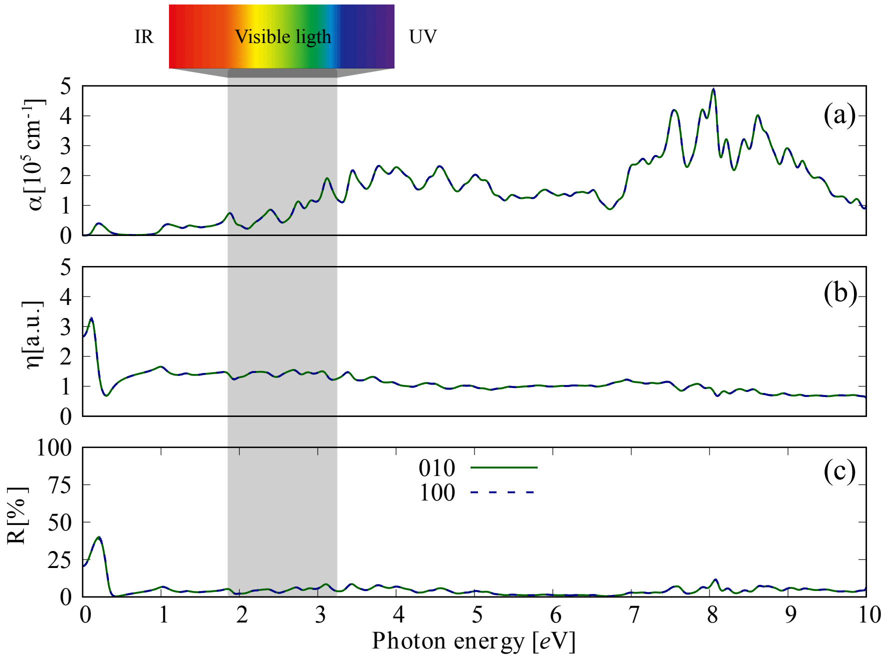

Figure S2: (a) optical absorption, (b) refractive index, and (c) reflectivity index calculated for a polarized light oriented along the x- (100) and y-direction (010). Absorption coefficient, reaching up to $5 \cdot 10^4$ cm^{-1}, highlights ISi's metallic nature. Prominent absorption peaks in the UV spectrum, with the highest around 4.5 eV, indicate unique electronic transitions compared to silicene's peak at 2.9 eV. The multiple absorption peaks for photon energies above 4.0 eV suggest ISi's potential as a UV detector and absorber, similar to silicene. The optical characteristics of ISi within the plane are isotropic and have lower intensity but exhibit similar features when compared to light polarized perpendicular to the plane of the nanomaterial.
